# The Evaluation of Diagnostic, Prognostic, and Predictive Role of Hematologic Inflammatory Indices NLR, PLR, and LMR in Common Solid Tumors

**DOI:** 10.1002/cnr2.70407

**Published:** 2025-11-27

**Authors:** Fatemeh Arianmanesh, Saeede Bagheri, Mohammad Ali Karimi, Saba Izadi, Mohammad Hossein Ahmadi

**Affiliations:** ^1^ Department of Hematology and Blood Banking, Faculty of Medicine Mashhad University of Medical Sciences Mashhad Iran; ^2^ Student Research Committee, Department of Hematology and Blood Banking, School of Allied Medical Sciences Shahid Beheshti University of Medical Sciences Tehran Iran; ^3^ Department of Laboratory Sciences, School of Paramedical Sciences Mashhad University of Medical Sciences Mashhad Iran

**Keywords:** breast cancer, colon cancer, lung cancer, monocyte‐to‐lymphocyte ratio, neutrophil‐to‐lymphocyte ratio, platelet‐to‐lymphocyte ratio, prostate cancer

## Abstract

**Background:**

Solid tumors are one of the leading causes of cancer‐related deaths. Measurable combined inflammatory markers in the blood, which indicate the inflammatory response, play a crucial role in managing patients with malignancies. These markers have been validated as less invasive, practical, and cost‐effective tools in the clinical decision‐making process. The aim of this study is to evaluate the predictive value of inflammatory markers based on complete blood count (CBC), including neutrophil‐to‐lymphocyte ratio (NLR), platelet‐to‐lymphocyte ratio (PLR), and lymphocyte‐to‐monocyte ratio (LMR) in major solid tumors, including breast, lung, colorectal, and prostate cancers, due to their high prevalence and strong evidence base.

**Recent Findings:**

In this study, studies from 2015 to June 2025 were searched in Google Scholar, PubMed, and Scopus using keywords such as neutrophil‐to‐lymphocyte ratio, platelet‐to‐lymphocyte ratio, monocyte‐to‐lymphocyte ratio, prognosis value, predictive value, diagnosis value, lung cancer, colon cancer, prostate cancer, and breast cancer. Findings indicate that elevated NLR and PLR, alongside reduced LMR, are commonly associated with advanced disease, poorer survival, and diminished response to treatment, though the strength of evidence varies by cancer type. Limitations across studies include retrospective design, inconsistent cut‐off values, and confounding factors such as comorbidities and treatment regimens.

**Conclusion:**

Current evidence suggests that NLR, PLR, and LMR have significant potential as accessible biomarkers for risk stratification and treatment monitoring in common solid tumors. However, lack of standardization in methodology and cut‐off definitions limits their widespread clinical implementation. High‐quality prospective studies are needed to establish unified thresholds and clarify their role alongside established biomarkers.

## 1 Introduction

Cancer is one of the leading causes of mortality worldwide. The four most commonly diagnosed cancers across both sexes worldwide are lung cancer (12.4%), female breast cancer (BC) (11.6%), colon cancer (CC) (9.6%), and prostate cancer (PCa) (7.3%), which highlights the impact of solid tumors on global cancer statistics and the overall health burden [1, 2].

Inflammation is defined as one of the hallmarks of cancer. Evidence suggests that, in addition to tumor characteristics, the inflammatory response also plays a crucial role in cancer growth and progression. Therefore, assessing the level of inflammation can be useful in the diagnosis, prognostication, and prediction of various malignancies [3, 4]. Tumor‐associated inflammation involves host‐derived cytokines, tumor‐derived cytokines, immune cells, and inflammatory mediators, which can be measured using blood cell markers such as lymphocytes, monocytes, neutrophils, and platelets [5, 6]. In recent years, composite inflammatory indices such as the neutrophil‐to‐lymphocyte ratio (NLR), platelet‐to‐lymphocyte ratio (PLR), and lymphocyte‐to‐monocyte ratio (LMR) have attracted considerable attention due to their accessibility, non‐invasive nature, cost‐effectiveness, and potential utility in evaluating systemic inflammation and immune status in cancer patients, based on routine complete blood count (CBC) tests [7, 8, 9, 10, 11, 12, 13, 14]. Compared to individual hematological parameters, composite inflammatory markers, such as NLR, PLR, and LMR, offer superior value in patient assessment owing to their enhanced sensitivity and stability [15]. These markers have been investigated across various tumor types [16, 17, 18, 19]; however, given the vast number of studies and the heterogeneity of cancers, a comprehensive analysis of all malignancies is beyond the scope of this article. Therefore, we focused on the most prevalent solid cancers with a larger body of published evidence, with the aim of addressing other solid tumors in future studies.

This review is organized according to cancer type (breast, lung, colorectal, and prostate). For each cancer, the available evidence on the role of systemic inflammatory markers (NLR, PLR, and LMR) is presented in prognostic, predictive, and diagnostic contexts, respectively. This structure allows clear within‐cancer comparisons of prognostic, predictive, and diagnostic roles, while enabling the integration of cancer‐specific biological details that would be difficult to contextualize in a purely biomarker‐based framework.

## 2 Inflammatory Cells and Hematological Biomarkers in the Tumor Microenvironment

Macrophages, neutrophils, and platelets in the chronically inflamed tumor microenvironment secrete cytokines, proteases, angiogenic factors, and chemokines. These cells play crucial roles in tumor initiation and progression, as extensively documented in solid tumors such as lung, breast, colorectal, and prostate cancers [20].

Neutrophils are key components of innate and adaptive immune systems, act as first responders to tissue injury and play a central role in inflammatory processes. Persistent neutrophil infiltration during chronic inflammation can promote tumorigenesis. Neutrophils are highly adaptable and can alter their phenotype in response to tumor‐derived or inflamed tissue signals [21, 22]. Tumor‐associated neutrophils (TANs) and polymorphonuclear myeloid‐derived suppressor cells (PMN‐MDSCs) represent two major neutrophil populations with specialized activation states that arise in response to tumor‐derived factors and inflammatory cues within the tumor microenvironment, acquiring immunosuppressive functions. They expand systemically in cancer and are associated with poor prognosis, metastasis, and resistance to immunotherapy [21, 22]. Both subsets exert immunosuppressive effects by producing arginase‐1 (ARG1) and inducible nitric oxide synthase (iNOS), leading to impaired CD8^+^ T cell activity. Moreover, neutrophils enhance angiogenesis and metastasis by releasing tumor necrosis factor‐alpha (TNF‐α) and vascular endothelial growth factor (VEGF) [23, 24]. Monocytes, either directly or through differentiation into dendritic cells or tumor‐associated macrophages (TAMs), contribute to tumor growth by enhancing VEGF production, releasing IL‐6 and IL‐1, and suppressing cytotoxic T cell responses via IL‐10 and transforming growth factor‐beta (TGF‐β) [25]. Activated platelets also facilitate tumor progression by releasing VEGF and shielding cancer cells from natural killer (NK) cell‐mediated immunity. Elevated platelet counts often indicate a worse clinical outcome [26]. Lymphocytes, particularly tumor‐infiltrating CD8+ T cells supported by CD4+ T cells (via IL‐2 and interferon‐gamma), are essential for anti‐tumor immunity. Their reduction is associated with poor prognosis [27].

Hematological ratios such as NLR, LMR, and PLR have emerged as indicators of systemic inflammation and immune status in cancer. NLR, calculated as absolute neutrophil count (ANC) divided by absolute lymphocyte count (ALC), is considered a marker of systemic inflammation. A high NLR may indicate the expansion of immunosuppressive granulocytic populations, including PMN‐MDSCs. Although this association is not specific, it suggests that NLR partly reflects immune suppression beyond general inflammation. LMR, defined as ALC divided by absolute monocyte count (AMC), indicates the balance between anti‐tumor lymphocytes and tumor‐supportive monocytes; lower LMR suggests higher tumor burden. PLR, calculated as platelet count divided by ALC, combines the effects of thrombocytosis and lymphopenia, with elevated values correlating with increased tumor aggressiveness and worse outcomes [28, 29].

While classical inflammatory markers such as C‐reactive protein (CRP) reflect the acute humoral protein response, hematological ratios including NLR, PLR, and LMR provide cell‐based measures of the immune–inflammatory balance. These indices capture shifts between innate and adaptive immunity, thereby offering complementary but distinct information compared to CRP [30, 31].

**FIGURE 1:**
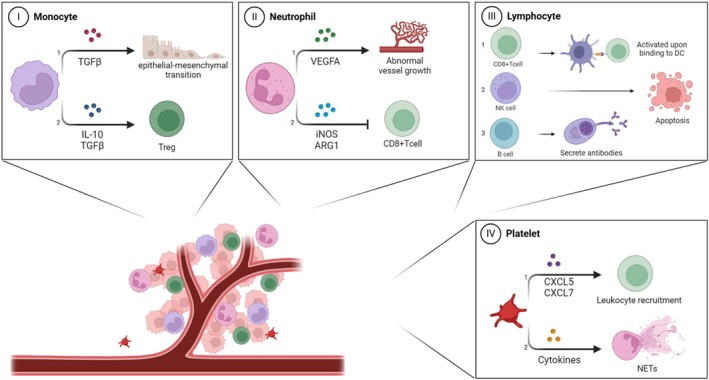
The role of blood cells in the tumor microenvironment. (I) Monocytes, either directly or through differentiation into dendritic cells or tumor‐associated macrophages (TAMs), promote: 1. epithelial‐mesenchymal transition (EMT) through TGFβ, increasing metastasis; and 2. the secretion of IL‐10 and TGFβ, which fosters regulatory T cells (Tregs) that inhibit effector T lymphocyte activity. (II) Neutrophils: 1. promote angiogenesis by releasing various pro‐angiogenic factors, including vascular endothelial growth factor A (VEGFA); and 2. support tumor growth by inhibiting CD8+ T cells. (III) Lymphocyte subsets: 1. Naïve CD8+ T cells activate by binding to antigen‐presenting dendritic cells in lymph nodes, then induce apoptosis in tumor cells as effector T cells; 2. NK cells identify tumor cells as “non‐self” and induce apoptosis; 3. B cells secrete tumor antigen‐specific IgG antibodies that trigger apoptotic responses upon binding to tumors. (IV) Platelets: 1. Activated platelets release pro‐inflammatory factors like CXCL5 and CXCL7, aiding leukocyte recruitment; 2. They promote the formation of neutrophil extracellular traps (NETs) through cytokine release, enhancing metastasis and thrombosis. Created with BioRender.com.

Overall, these ratios reflect the interplay between the tumor microenvironment and the host immune system and hold diagnostic, prognostic, and predictive value in oncology (Figure 1).

## 3 Clinical Evidence Across Major Solid Tumors

Given the heterogeneity of tumor biology and clinical settings, the evidence for NLR, PLR, and LMR is discussed separately for each major solid tumor, covering prognostic, predictive, and diagnostic implications.

### 3.1 Lung Cancer

#### 3.1.1 Introduction

Lung cancer is the most common malignant tumor and the leading cause of cancer‐related death globally among men. In women, it ranks third in incidence after BC and colorectal cancer (CRC) and second in mortality after BC [32, 33]. This type of cancer has a high mortality rate due to its late‐stage diagnosis. Tobacco use accounts for 90% of cases, though only a fraction of smokers develops the disease, suggesting additional environmental and genetic factors [34]. Despite various treatment modalities such as surgery, chemotherapy, radiotherapy, and targeted therapies, the prognosis remains poor, with a 5‐year survival rate of approximately 17% [35]. Routine prognostic factors include disease stage, age, gender, tobacco use, nutritional status, genetic mutations, body mass index (BMI), and treatment‐related factors [32, 36, 37]. Current diagnostic methods, such as examining lung lavages, CT scans, and chest x‐rays, are either invasive or involve radiation [32, 38]. Therefore, it is essential to find a reliable diagnostic and prognostic approach with minimal side effects for lung cancer.

#### 3.1.2 Subtypes and Classification

Lung cancer is histologically classified into two main groups: Non‐small cell lung cancer (NSCLC) and small cell lung cancer (SCLC). SCLC originates from neuroendocrine cells in the bronchial and epithelial base. This malignancy can occur either as a standalone condition or in combination with NSCLC, accounting for 15%–20% of primary lung cancers [39]. NSCLC involves epithelial cells from the central bronchi to the terminal air sacs and comprises approximately 80% of primary lung cancers [39, 40, 41]. Lung cancer staging includes Stage I (localized tumor), Stage II (spread to nearby lymph nodes), Stage IIIa‐b (regional spread with or without invasion of vital structures), and Stage IV (distant metastasis outside the thorax) [42]. The treatment of NSCLC and SCLC patients depends on the stage of the disease. Patients with early‐stage disease undergo surgery along with chemotherapy. In contrast, patients who are ineligible for surgery are treated with conventional or stereotactic radiotherapy [43, 44].

#### 3.1.3 Clinical Utility of Hematological Markers

Hematological markers derived from routine blood counts, including NLR, PLR, and LMR, have been explored for their potential role in risk stratification, treatment prediction, and early detection in lung cancer.

##### 3.1.3.1 Prognostic Value

Hematological ratios have been extensively studied as prognostic indicators in lung cancer, and their associations with survival outcomes are summarized below.

###### 3.1.3.1.1 Neutrophil‐To‐Lymphocyte Ratio (NLR)

Immunotherapy: Recent promising results have been reported with immune checkpoint inhibitors (ICIs) targeting cytotoxic T‐lymphocyte‐associated antigen 4 (CTLA‐4) and programmed death‐1 (PD‐1) in lung cancer patients. However, this treatment is expensive. Therefore, there is a need for reliable predictive and prognostic markers to identify a relatively small subset of patients who may benefit from long‐term treatment. A study on the effects of immunotherapy in NSCLC demonstrated that an increased NLR at six weeks into treatment was associated with worse overall survival (OS) and progression‐free survival (PFS), indicating a poor prognostic value for the individual [45]. Another study found that a low NLR (< 5) before treatment with nivolumab could predict a duration of OS that was twice as long compared to individuals with a high NLR (≥ 5) [46]. Similar findings were reported by Russo et al. They examined NSCLC patients treated with nivolumab at 14 cancer centers and concluded that patients with a high NLR (≥ 5) before treatment with nivolumab did not respond well to the therapy and should consider alternative treatments [47]. A recent study with a larger population than the Russo study confirmed the prognostic significance of baseline NLR (cut‐off = 3.86) in NSCLC patients treated with ICIs. This dual‐center analysis demonstrated that low baseline NLR prior to the initiation of immunotherapy was an independent factor for poorer OS and PFS. It also has a stronger predictive value for OS than PD‐L1 expression [48]. Additionally, another study found that a high NLR (> 5) prior to treatment in patients receiving immunotherapy as a second‐line treatment was directly associated with a higher risk of rapid disease progression and lower OS in patients with advanced NSCLC [41].

Radiotherapy: There have been limited studies investigating changes in NLR (ΔNLR = maximum NLR after surgery—NLR before surgery) and considering ΔNLR as a prognostic marker. A study conducted in China showed a direct association between a high ΔNLR (≥ 2.09) and poor outcomes in OS and PFS for patients with stage III NSCLC treated with conventionally fractionated radiotherapy. In contrast, a high NLR (≥ 6.48) after treatment solely indicated PFS state and was correlated with a shorter duration [49]. This supports the idea that dynamic changes in NLR, not just baseline levels, could reflect the systemic inflammatory response to treatment and provide clinicians with a real‐time prognostic tool.

Surgery: In a study evaluating PLR, NLR, and the Prognostic Nutritional Index (PNI), it was found that these biomarkers had significant prognostic value in surgically treated NSCLC patients, with NLR showing superiority over the other two indices. Patients with preoperative NLR ≥ 2.3 had lower rates of recurrence‐free survival (RFS). Furthermore, higher preoperative NLR was indicative of more advanced tumors, poorer differentiation, and greater invasiveness [50]. However, some surgical studies report inconsistent results. In a conflicting study with a smaller patient group focusing on NLR, PLR, and LMR in lung cancer patients undergoing radical surgery, it was concluded that NLR, as an accessible inflammatory marker, had limited utility in assessing disease progression [51]. possibly due to heterogeneity in patient populations, tumor stages, or lack of uniform NLR cut‐offs.

###### 3.1.3.1.2 Platelet‐To‐Lymphocyte Ratio (PLR)

Immunotherapy: Some studies have shown that PLR, like NLR, has significant prognostic value in the course of lung cancer and can be used to assess disease level and guide treatment. A high PLR before treatment in NSCLC indicates a poor prognosis for patients undergoing treatment with nivolumab, with higher PLR correlating with poorer OS compared to those with lower PLR [47].

Radiotherapy: In 544 SCLC patients undergoing radiotherapy, PLR showed a desirable prognostic value for the disease course in SCLC prior to treatment [36]. In contrast, a more recent study with a smaller sample size (167 patients) by Sun et al. on SCLC patients treated with radiotherapy indicated that, unlike high NLR (cut‐off = 3.06), high PLR (cut‐off = 168.03) had little correlation with OS and PFS. However, assessing the changes in NLR and PLR during treatment may enhance the usefulness of these markers as predictive biomarkers [52]. Due to the discrepancies regarding the diagnostic value of PLR for NSCLC, large‐scale, prospective, and multicenter studies are required.

Chemotherapy: In another study conducted by Yang et al. in 2022 in China, high baseline PLR (≥ 255.5) and NLR (≥ 3.7) in NSCLC patients undergoing chemotherapy combined with bevacizumab were inversely associated with OS and PFS [53]. A study involving a larger group of patients (438 patients) using chemotherapy or chemoradiotherapy (CRT) for treatment examined the prognostic utility of three markers: PLR (cut‐off = 150 and 250), NLR (cut‐off = 4 and 5), and LMR (cut‐off = 2.64 and 4.19) in SCLC patients. The study revealed that a low NLR was associated with a better prognosis in patients with extensive‐stage disease (ED), while PLR and LMR had no specific utility for either the ED or limited‐stage disease (LD) groups [54].

Surgery: Huang et al. conducted a study involving 254 NSCLC patients who underwent surgery and found that patients with PLR ≥ 122.22 had significantly worse OS compared to those with PLR < 122.22. They concluded that PLR has a superior prognostic value for treatment outcomes and cancer progression compared to other inflammatory markers. Additionally, PLR may be useful for classifying disease levels and guiding postoperative treatment [55]. Łochowski et al. noted the association between high PLR and survival chances in NSCLC patients with an average age of 63.6. These patients underwent chemotherapy and surgery, and the study indicated that OS for those with high PLR (> 144) would be less than two years [56].

Overall, most studies suggest that a high pre‐treatment PLR is associated with worse OS and may have prognostic value in lung cancer. However, its predictive value appears inconsistent across treatment types and lung cancer subtypes. Variations in cut‐off definitions and patient characteristics could partly explain these inconsistencies.

###### 3.1.3.1.3 Lymphocyte‐To‐Monocyte Ratio (LMR)

Chemotherapy: This inflammatory marker, unlike the NLR and PLR, has poor prognostic value under reduced conditions. A study aimed at evaluating the utility of all three indices in lung cancer patients found that after chemotherapy combined with immunotherapy, a notably low baseline LMR (< 3) directly correlated with higher OS and PFS [53]. Similarly, patients at stage IIIB‐IV NSCLC undergoing chemotherapy, low LMR (≤ 3.73) predicted poor OS and PFS, suggesting prognostic relevance in advanced disease [57].

Surgery: In a study by Ramos et al., which evaluated NSCLC patients undergoing radical surgery for 52 months, LMR (cut‐off = 2.5) was identified as a prognostic marker among the three markers (LMR, PLR, NLR) that correlated with longer PFS and OS [51]. Conversely, a study reported that LMR and NLR, unlike PLR, do not serve as practical prognostic markers for OS in lung cancer patients, possibly due to differences in blood sample collection timing and variable classification methods compared to similar studies. This study involved 544 NSCLC patients receiving radiotherapy [36].

##### 3.1.3.2 Predictive Value

Beyond prognosis, these markers have also been evaluated for their role in predicting treatment response, as outlined in the following subsections.

###### 3.1.3.2.1 NLR

Immunotherapy: Several studies have investigated the predictive role of NLR in NSCLC patients undergoing ICI therapy. A Japanese study found that a low NLR (< 2.86) before immunotherapy in patients with NSCLC was associated with a lower risk of immune‐related adverse events (irAEs). The study also indicated that NLR could be used to effectively manage irAEs before they occur [58]. Another study noted that a high NLR (cut‐off = 5) predicted shorter OS, but there was no specific correlation with PFS. While in patients with low NLR (< 4), the efficacy of ICIs in the presence of steroid medications was reduced [59]. In a study conducted by Lim et al. on patients with advanced NSCLC, it was found that a primary change of ≥ 1 in the derived neutrophil‐to‐lymphocyte ratio (d‐NLR) had good predictive value for early disease progression [60]. These findings suggest that NLR, both at baseline and dynamically, may help anticipate immunotherapy‐related toxicity and early disease progression.

Targeted Therapy: Research by Xu et al. showed that a higher NLR (≥ 2.57) in advanced‐stage NSCLC receiving tyrosine kinase inhibitors (TKIs) was linked to a significantly lower disease control rate (DCR) at 8 and 10 months after treatment with TKIs. It was concluded that a high NLR could serve as a predictive marker for the therapeutic effects of TKIs in patients with advanced‐stage NSCLC [61].

A large cohort study (*n* = 658) linked NSCLC patients with high NLR (≥ 5) to lower OS and time to treatment failure (TTF) [62]. These studies reinforce NLR's potential as a general predictor of poor response, although its predictive utility may vary by treatment type.

###### 3.1.3.2.2 PLR

Immunotherapy: Data from 62 NSCLC patients receiving nivolumab or docetaxel were analyzed by Russo et al., who determined that a high PLR (≥ 160) was associated with a low overall response rate (ORR) in both groups. This inflammatory index may be useful for assessing baseline status in patients eligible for immunotherapy [63]. In another clinical study involving patients with metastatic NSCLC treated with nivolumab, high pre‐treatment PLR and NLR were associated with reduced survival and lower response rates. These markers demonstrated acceptable predictive performance for 10‐month survival and treatment response, highlighting their combined prognostic and predictive value in the context of immunotherapy [64].

Targeted Therapy: In a study by Chen et al. involving 152 patients with advanced NSCLC who received anlotinib, it was found that high NLR (> 3.41) and PLR (> 205.63) before treatment were associated with poor DCR [65]. Although this study demonstrated an association between elevated NLR and PLR and poor treatment response, its retrospective design and relatively limited sample size warrant confirmation through larger, prospective studies.

###### 3.1.3.2.3 LMR

Chemotherapy: Besides the prognostic value of LMR for patient survival, this index can also be used to predict patients' responses to treatment. A study examining blood indices in NSCLC patients before chemotherapy indicated that a high LMR (> 3.73) was directly associated with treatment effectiveness in patients [57]. Further prospective validation is needed due to the limited sample size.

Targeted Therapy: An analysis of 53 patients with advanced SCLC receiving anlotinib showed that increased NLR after treatment, high pre‐treatment PLR (> 240.56), and low pre‐treatment LMR (≤ 1.61) were independently associated with a low DCR and high PFS, while no correlation was found with OS [66]. Although LMR showed predictive value in this study, its lack of association with OS may limit its clinical applicability.

##### 3.1.3.3 Diagnostic Value

Several studies have shown that NLR and PLR with specific threshold values can be used in the diagnosis of NSCLC. These markers can serve as alternatives to invasive diagnostic methods, helping to avoid unnecessary expenses and procedures while saving time. A study involving 245 participants concluded that NLR (cut‐off = 2.14) can be used as a diagnostic tool for NSCLC, especially when combined with red blood cell distribution width (RDW), hemoglobin‐to‐red blood cell distribution width ratio (HRR), and carcinoembryonic antigen (CEA) [67]. In the study by Zhu et al., it was noted that in the lung cancer patient group, an increase in NLR (cut‐off = 2.14) was associated with a rise in total white blood cell (WBC) count, while no changes were observed in PLR (cut‐off = 149.95), which indicates the lower diagnostic utility of PLR compared to NLR. Furthermore, it was found that both NLR and PLR individually possess moderate diagnostic capabilities, and this diagnostic value increases when these two parameters are combined [14].

### 3.2 Breast Cancer

#### 3.2.1 Introduction

BC is a heterogeneous disease influenced by both genetic and non‐genetic factors. According to recent global statistics, BC has become the most common type of cancer in women and the second most common cancer worldwide. Breast tumors typically originate from the proliferation of ducts and can transform into benign tumors or even metastatic carcinomas after continuous stimulation by various carcinogenic factors [68, 69]. The management of BC includes surgery, radiation therapy, chemotherapy, endocrine therapy, and targeted therapies (such as endocrine therapy (ET) and anti‐HER2 treatment). Biopsy is the primary approach for diagnosing BC; however, its practicality for screening is limited due to its invasive nature. Furthermore, emerging screening methods, such as circulating tumor cell (CTC) measurement, are constrained by high costs and equipment requirements. Due to the risk of recurrence and metastasis, the prognosis for patients with BC remains unfavorable, and additional predictive and prognostic biomarkers are needed to optimize treatment strategies for individual patients [70, 71, 72, 73, 74].

#### 3.2.2 Subtypes and Classification

Immunohistochemical classification based on the expression of estrogen receptors (ER), progesterone receptors (PR), and human epidermal growth factor receptor 2 (HER2) defines four subtypes of BC: Luminal A, Luminal B, HER2‐positive, and triple‐negative. Luminal A tumors are clinically low‐grade and have the best prognosis with lower recurrence. Luminal B tumors have a higher grade and worse prognosis. The HER2‐positive group represents 10%–15% of BCs and is characterized by high HER2 expression and the absence of ER and PR. Triple‐negative breast cancer (TNBC) is defined as negative for ER, PR, and HER2 and constitutes about 20% of all BCs. TNBC is marked by its aggressive nature, early recurrence, and a greater tendency to occur at advanced stages [75].

#### 3.2.3 Clinical Utility of Hematological Markers

These ratios, calculated from standard CBC parameters, are investigated for multiple clinical purposes in BC, ranging from prognosis and treatment prediction to diagnostic support.

##### 3.2.3.1 Prognostic Value

Although some predictive factors and prognostic indicators are available, such as hormone receptor status, tumor stage (localized vs. metastatic), and molecular subtypes (luminal, HER2‐positive, or triple‐negative breast cancer), most of these factors are typically obtained through core biopsies and with the aid of molecular techniques that are invasive and expensive. The accuracy of these prognostic indicators has also been reported to be unsatisfactory. Therefore, easier and more efficient prognostic parameters are needed to guide optimal treatment [70, 71].

###### 3.2.3.1.1 NLR

Neoadjuvant chemotherapy (NAC) is a common treatment approach for patients with BC. In many studies, a complete pathological response (pCR) to NAC has been associated with longer disease‐free survival (DFS) and OS for patients. In a study by Li et al., the medical records of 282 patients with early‐stage BC who received NAC were reviewed. This study found that a low NLR (< 1.8) before treatment in the peripheral blood of BC patients predicted a higher pCR and better OS after NAC [76]. Another study of 358 patients with advanced localized TNBC receiving NAC found that a high baseline NLR (> 3.16) was associated with poor survival in these patients [77]. Alshamsan et al. also analyzed data from 465 patients with locally advanced BC undergoing NAC and confirmed that a low NLR (≤ 2.2) was associated with improved long‐term survival (DFS and OS), particularly in patients with TNBC. With its large sample size, this study provides credible evidence supporting the prognostic value of NLR, although limitations such as its retrospective design and lack of tumor tissue analysis should be noted [78].

HER2‐targeted therapy: One of the major advancements in BC treatment has been the development of trastuzumab, a HER2 antibody that has significantly improved outcomes for HER2‐positive patients. Tiainen et al. evaluated patients with primary BC, especially those with HER2+ tumors, and found that high NLR (≥ 2.2) was associated with poorer survival. However, this association was not observed in patients receiving adjuvant trastuzumab, suggesting that trastuzumab may offset the negative prognostic effects of systemic inflammation. Despite limitations such as the retrospective design and small subgroup sizes, the study proposes an important hypothesis on the interaction between immunity and targeted therapy in HER2+ breast cancer [79]. In a study with shorter follow‐up (20 months vs. 10 years) of 843 patients with HER2‐positive breast cancer, NLR was not prognostic without trastuzumab but was associated with better DFS when baseline NLR was low (cut‐off = 1.830) in those receiving one year of adjuvant trastuzumab. Differences in cut‐off, follow‐up, or additional treatments may explain the discrepancy. Still, its independent prognostic value supports NLR's role in managing HER2+ patients [80].

Surgery: Some evidence supports the prognostic value of NLR in surgically treated patients. In the study by Inoue et al., data were collected from 397 patients with oligometastatic breast cancer (OMD) who had undergone primary breast surgery and experienced a relapse during a follow‐up period of 59 months. The results of this study indicated that a low NLR (cut‐off = 2.52) is an independent prognostic factor for better OS in OMD patients [81]. Kim et al. included patients with TNBC who underwent surgery and concluded that changes in NLR could reflect the prognosis of TNBC patients [82]. However, conflicting results also exist; Sifón et al. analyzed 791 patients with early‐stage invasive BC who underwent surgery and concluded that NLR does not appear to be an independent prognostic factor for recurrence in this population. Given the inclusion of patients across different disease subtypes (e.g., TNBC, OMD, early‐stage) and varying treatment approaches (surgery alone or combined with chemotherapy and immunotherapy), generalizing the findings remains challenging [83].

###### 3.2.3.1.2 PLR

This section aims to evaluate the prognostic value of PLR in BC patients and its potential utility as an independent clinical marker, particularly in the context of NAC or surgical treatment.

Neoadjuvant chemotherapy: The prognostic value of NLR and PLR in TNBC patients undergoing NAC was evaluated in a 2024 study, which found that lower NLR and PLR levels were associated with longer event‐free survival (EFS) and OS. However, limitations included the use of median‐based cut‐off values and a small sample size [84]. Another study reported a positive correlation between PLR and intratumoral Treg cell populations, suggesting that elevated PLR may reflect immunosuppression. The study population consisted of TNBC patients, some of whom received NAC. Additionally, high tumor‐infiltrating lymphocyte (TIL) levels and low PLR were associated with the highest rates of distant metastasis‐free survival (MFS) and OS [85].

Surgery: Cho et al. reviewed the clinical and pathological records of 661 patients with invasive BC who underwent surgery. The results showed that a high PLR (> 185.5) independently predicted poor disease‐specific survival (DSS) and DFS in BC patients. However, no correlation was observed with tumor stage or lymph node metastasis [86]. Additionally, a retrospective evaluation by Xu et al. of 508 BC patients treated surgically, suggested that a higher PLR (> 150.65) was associated with worse DFS [87].

Two meta‐analyses investigating the prognostic significance of PLR in BC have reported conflicting results. One study found that elevated PLR was associated with poorer DFS and OS among BC patients [88]. However, another meta‐analysis reported no significant association between PLR and OS in BC, in contrast to cancers such as colorectal, gastric, esophageal, hepatic, pancreatic, and ovarian, where high PLR was linked to worse outcomes [89]. Given that PLR results in both univariate and multivariate models were only marginally significant, further studies are needed to clarify the prognostic value of PLR in BC.

###### 3.2.3.1.3 LMR

Recent studies have reported that the LMR, which reflects systemic inflammation, is associated with the survival of BC patients.

Chemotherapy and surgery: In another study involving 232 patients with invasive ductal carcinoma, of whom 96% received either chemotherapy or NAC, a low LMR (< 5.3) was also associated with poor OS and DFS [90]. A group of 150 bc patients treated with NAC followed by surgery was analyzed by Marín Hernández et al. Those with a high LMR (≥ 5.46) and a low NLR (< 3.33) had a lower rate of recurrence [91]. A study with a larger sample size (440 patients) and a longer follow‐up period (72 months) in BC patients who underwent surgery followed by standard treatment (NAC and radiotherapy) showed that a higher LMR (≥ 4.85) was associated with longer average DFS [92]. A few studies have suggested that a low LMR may serve as a better prognostic indicator than NLR for predicting poor DFS in BC patients [93, 94].

##### 3.2.3.2 Predictive Value

Inflammatory ratios have been widely analyzed in BC, particularly in relation to predictors of response.

###### 3.2.3.2.1 NLR

Chemotherapy: Several studies have explored the role of changes in serum NLR related to treatment outcomes in BC during chemotherapy. An analysis of data from 346 BC patients undergoing NAC over a 5‐year period in the study by Zhu et al. showed that among patients who achieved a pCR after NAC, those with a low NLR (< 1.695) had a significantly better pCR rate compared to those with a high NLR (≥ 1.695) [95]. Despite the strengths of this study, such as the uniform chemotherapy regimen and control of confounding variables (e.g., no prior invasive procedures before testing), the lack of long‐term outcome assessment and survival data limits the interpretation of its prognostic implications. Another study evaluating eribulin in patients with metastatic breast cancer reported that a low baseline NLR (< 3) was significantly associated with improved PFS in these patients [96]. However, another study involving 959 patients across various BC subtypes found no significant association between NLR and prognostic indicators such as pCR or OS. This discrepancy may be attributed to differences in statistical analysis (using NLR as a continuous variable) and the heterogeneity of the patient population in terms of tumor subtypes and treatment modalities (neoadjuvant vs. adjuvant) [97].

###### 3.2.3.2.2 PLR

Chemotherapy: Some studies suggest a potential predictive role for PLR in identifying BC patients who are more likely to achieve a pCR following NAC. In a study involving 95 bc patients who received neoadjuvant therapy before surgery, researchers Yang et al. demonstrated that low levels of NLR (< 2.46), PLR (< 118.78), and the Systemic Immune‐Inflammation Index (SII) (< 403.20) prior to treatment could serve as predictors for a high pCR rate in these patients [98]. Additionally, in a study by Kaytaz Tekyol et al., 150 bc patients who were initially prescribed NAC and subsequently underwent surgery were evaluated. Patients with low PLR levels (< 150) exhibited high chemotherapy sensitivity independent of molecular subgroups in BC treatment with NAC [99]. Although both studies suggest that lower PLR values may predict better pCR in BC patients undergoing NAC, differences in cut‐off points and study design (e.g., retrospective nature, limited sample size) highlight the need for standardized prospective validation.

###### 3.2.3.2.3 LMR

Surgery and chemotherapy: A cohort of 542 patients with advanced BC (T3/T4 and/or N2/N3) who received NAC followed by surgery was studied. The results indicated that higher LMR levels (≥ 4.25) were significantly associated with a better ORR in patients undergoing NAC [100]. Peng et al. included a group of BC patients treated with NAC and subsequent surgery in their study, finding that a low LMR (< 6.1) is considered an independent predictor of NAC efficacy in BC patients [101]. The findings of both studies support the potential of LMR as a clinical predictive marker. However, differences in threshold values and study designs (such as variations in disease stage or treatment regimens) should also be taken into account.

##### 3.2.3.3 Diagnostic Value

Improvements in BC screening and neoadjuvant therapy have led to a reduction in mortality rates. While treatment increases survival when administered at the right time, early diagnosis provides the best survival rates. Therefore, new diagnostic markers are essential for reducing mortality and complications. In a large study involving 808 bc patients and 2424 healthy controls, the average NLR in BC patients (2.28) was significantly higher compared to the normal individuals (2.04), suggesting a possible diagnostic value for this index [101]. A notable diagnostic application was reported by Velidedeoglu et al., who compared 50 bc patients, 50 healthy female volunteers, and 55 patients with idiopathic granulomatous mastitis (IGM), which is a benign condition often misdiagnosed as BC. There was a statistically significant difference between the two patient groups; IGM patients had higher levels of fibrinogen (> 345.0 mg/dL), fibrinogen/albumin (Fib/Alb) ratio (> 82.79), CRP (> 3.625 mg/L), and NLR (> 2.285) compared to BC patients, demonstrating strong potential as practical guides for differentiating BC from IGM [102]. Morkavuk et al. reported that in a study of 202 patients diagnosed with early‐stage BC, the risk of having a positive sentinel lymph node was 0.43 times higher in patients with high PLR (> 139.45) compared to those with low PLR (< 139.45). Interestingly, the diagnostic performance of PLR was higher than that of standard imaging tools, suggesting its potential as a supportive diagnostic adjunct in staging rather than initial diagnosis [103]. Furthermore, in a 2025 study assessing the diagnostic value of several indices (PLR, NLR, LMR, and PNI) in patients with BC, PLR showed a significant difference between patients and the control group. However, the lack of significant differences in the other indices, the absence of pathological data (such as tumor type or grade), and the small sample size limit the validity and generalizability of the findings [104].

### 3.3 Colorectal Cancer

#### 3.3.1 Introduction

CRC is the most common gastrointestinal cancer, the third most prevalent cancer in men and the second in women worldwide, accounting for over 1.9 million new cases in 2020. It is also the second leading cause of cancer‐related deaths [105]. CRC is a multifactorial disease involving genetic, environmental, and lifestyle factors, often progressing from benign polyps to adenomas, which can ultimately lead to carcinoma. The main treatment is surgical, which is complemented by chemotherapy, radiotherapy, targeted molecular therapy, and immunotherapy [106]. Current conventional methods for diagnosing CRC include colonoscopy (the gold standard), fecal occult blood tests (FOBT), fecal immunochemical tests (FIT), and CEA or CA‐19‐9 (carbohydrate antigen‐19‐9), all of which have their specific limitations. Therefore, identifying a non‐invasive approach with high diagnostic and prognostic accuracy is essential [107].

#### 3.3.2 Subtypes and Classification

CRC can be categorized as 72% for CC and 28% for rectal cancer, although the incidence rates are generally reported together. More than 90% of colorectal carcinomas are adenocarcinomas originating from the epithelial cells of the colorectal mucosa. Most cases (about 95%) of CRC are sporadic, occurring due to random mutations in genes. Hereditary CRC (familial) is less common (about 5%); hereditary CRC patterns include hereditary non‐polyposis colorectal cancer (HNPCC), familial adenomatous polyposis (FAP), MYH‐associated polyposis (MAP), Peutz‐Jeghers syndrome (PJS), and juvenile polyposis syndrome (JPS) [108, 109]. The most commonly used staging system for CRC is the tumor‐node‐metastasis (TNM) system. This staging includes stages 0, I, II (with three subgroups IIA, IIB, and IIC), III (with three subgroups IIIA, IIIB, and IIIC), and IV [109, 110].

#### 3.3.3 Clinical Utility of Hematological Markers

Given their accessibility and cost‐effectiveness, NLR, PLR, and LMR have attracted attention as potential tools for prognosis, therapy response prediction, and diagnostic assessment in CRC.

##### 3.3.3.1 Prognostic Value

Clinical stage, tumor histopathology, tumor localization, and the number of involved lymph nodes are widely used to predict the prognosis of CRC. However, current staging methods, such as fine‐needle biopsy and metastasis assessment, are invasive. Furthermore, most individuals with the same clinical stage and tumor differentiation may have different prognoses. Therefore, it is essential to identify more effective and less invasive predictive indicators for CRC stages [111, 112].

###### 3.3.3.1.1 NLR

Chemotherapy: Studies conducted on patients with metastatic CRC [113] and advanced colorectal cancer [114], receiving first‐line chemotherapy, revealed that a low NLR is associated with longer average OS. Consequently, measuring NLR can help predict survival outcomes in these patients.

Surgery: In a retrospective study involving 330 surgical CRC patients, it was found that high NLR (≥ 3.03) significantly affected survival in stage I–II CRC patients, which underscores the utility of NLR even in early‐stage disease, where traditional prognostic markers may be less discriminatory [115]. Another retrospective study involving 1052 surgical patients recommended preoperative NLR values ≥ 3.11 and LMR < 3.39 as independent prognostic parameters for response to CRT and a lower risk of lymph node metastasis [116]. Additionally, a study by Cui et al. involving 146 CRC patients found that both preoperative (cut‐off = 2.39) and postoperative (cut‐off = 2.96) NLR values were significantly associated with a poor prognosis, while changes in NLR (∆NLR) did not demonstrate a strong prognostic value [117]. These findings suggest that static pre‐ and post‐treatment NLR levels may be more informative than dynamic changes in assessing long‐term outcomes. Another large‐scale study involving 576 CRC patients at stages I–IV also demonstrated that those with lower pre‐NLR (cut‐off = 2.33) and post‐NLR (cut‐off = 2.06) had better OS; moreover, combining pre‐ and post‐operative NLR increased prognostic accuracy compared to using each parameter alone [118].

###### 3.3.3.1.2 PLR

The relationship between PLR levels and prognosis in patients with CRC has been investigated in various studies. In patients with advanced colorectal cancer undergoing palliative treatment, PLR emerged as an independent prognostic factor for survival time in CRC patients, with a high PLR (≥ 207.29) significantly associated with reduced survival, indicating its utility even in later stages of disease when therapeutic options are limited [119]. In a study analyzing 209 patients with oligometastatic colorectal cancer (OMCC), it was shown that pre‐treatment PLR (surgery, chemotherapy, radiotherapy, or targeted therapy) was an independent prognostic factor for survival, with high PLR (cut‐off = 208) significantly associated with worse OS and PFS [120].

Surgery: Abdallah et al. conducted a prospective study in Brazil and found that higher PLR (> 124) had significantly lower DFS compared to those with low PLR, patients with non‐metastatic colon cancer undergoing resection surgery, especially when evaluated alongside CTCs and clinical characteristics [121]. Similarly, Li et al. reported that preoperative PLR (cut‐off = 260) could serve as an independent prognostic indicator for OS in stage I–III colon cancer patients, with lower preoperative PLR significantly associated with better survival [115]. Xia et al. also found that rectal cancer patients undergoing surgical resection with stage T1‐2 cancer and high PLR (≥ 140) had much worse three‐year OS compared to those with low PLR (≤ 140) [122]. In a large retrospective study involving over 1000 patients with CRC, preoperative PLR and NLR were found to be associated with OS and DFS. Patients with a PLR ≥ 157.62 exhibited poorer prognoses. This study supports the important role of PLR as a reliable inflammatory marker for assessing long‐term prognosis in CRC patients, particularly in the context of anastomotic leakage [123].

###### 3.3.3.1.3 LMR

Chemotherapy: Ciocan et al. demonstrated an inverse association between low LMR values (cut‐off = 16.21) and advanced tumor stage and metastasis, linking it to worse outcomes regardless of age and gender [124].

Surgery: Several studies have confirmed the prognostic value of preoperative LMR in CRC patients. For instance, an analysis of 554 patients with advanced CC who underwent surgery showed that among stage III CC patients, OS was significantly better in the group with high LMR (≥ 2.77) compared to those with low LMR (< 2.77). Additionally, LMR may have a significant impact on the prognosis for patients with lymph node metastasis and may also assist in selecting post‐operative treatment options [125]. Similar findings were reported in patients undergoing surgery, where higher LMR (≥ 4.8) predicted better OS and DFS [126]. Further studies have confirmed the prognostic role of LMR (cut‐off = 1.67 and 2.4) in CC patients across both emergency and elective surgeries [127, 128]. However, despite consistent results, significant heterogeneity exists in the reported cut‐off values (ranging from 1.67 to 4.8), sample sizes, and surgical contexts (elective vs. emergency). Moreover, few studies integrated LMR with molecular or pathological tumor characteristics.

##### 3.3.3.2 Predictive Value

It has been established that LMR, PLR, and NLR reflect tumor size and cancer stage and can be utilized to predict treatment outcomes in patients with CRC [129, 130].

###### 3.3.3.2.1 NLR

Chemotherapy: In a study by Lasagna et al., which examined patients with metastatic colorectal cancer (mCRC) treated with panitumumab in combination with first‐line chemotherapy, it was found that a high baseline NLR (≥ 2.72) and low LMR (< 2.81) were predictors of poorer initial response to panitumumab treatment [131]. Compared to the study by Lasagna, which focused solely on NLR as a predictor of response to panitumumab, Jiang et al. simultaneously analyzed multiple inflammatory markers, including NLR, PLR, and SII. The study found that low inflammatory biomarker levels, including NLR (< 3.285), low PLR (< 171.45), and low SII (< 660.55), predicted a higher initial response rate to treatment in mCRC patients. These parameters also indicated longer PFS and OS for initial responders compared to non‐responders [132].

Surgery: A study by Nora et al. involving 116 patients with gastrointestinal malignancies found that increased NLR (cut‐off = 2.25) and PLR (cut‐off = 140) were associated with disease recurrence and progression after surgery. Importantly, patients whose NLR and PLR did not return to normal levels within three months post‐surgery were more likely to experience early recurrence or persistent disease [133]. Similarly, Fuss et al. found that in CRC patients undergoing surgery, high NLR (≥ 3) was significantly associated with an increased risk of complications, lower OS, and DFS [134]. Pereira et al. reported that NLR (cut‐off = 2.6) was higher in patients with T3–T4 tumors compared to T1–T2, confirming its potential as a predictive indicator for tumor invasion [135]. Finally, another study indicated that high NLR levels (cut‐off = 3.7) were associated with more advanced tumor stages and lymph node metastasis, reinforcing its value in preoperative assessment [112].

###### 3.3.3.2.2 PLR

Acikgoz et al. investigated 229 CRC patients and found that high PLR levels (> 196.5) were associated with a higher incidence of BRAF mutations and proximal colon tumors. Better OS, DFS, and treatment response were also observed in patients with low PLR compared to those with high PLR, suggesting that PLR may reflect both tumor biology and therapeutic outcomes [136].

Chemotherapy: In a study analyzing patients with stage II CRC who underwent surgical treatment, high PLR (> 130) predicted a greater benefit from chemotherapy, with improved cancer‐specific survival (CSS) [137]. Similarly, in a retrospective study of 396 rectal cancer patients, pre‐treatment NLR > 2.08 and PLR > 129.36 were associated with an increased risk of incomplete response to NAC [138]. These findings support the utility of this marker in selecting patients more or less likely to benefit from standard neoadjuvant chemoradiotherapy.

###### 3.3.3.2.3 LMR

Chemotherapy: In a study of 104 patients with unresectable mCRC undergoing palliative chemotherapy, normalization of LMR eight weeks after the start of chemotherapy was associated with improved OS. Additionally, DCRs were significantly higher in the high LMR group (cut‐off = 3.38), indicating that this parameter is a useful marker for predicting chemotherapy response in this patient category [139].

Surgery: Several studies suggest preoperative LMR may help predict recurrence and long‐term outcomes. In patients with stage II–III CRC and type II diabetes, low CA‐19‐9 and high LMR levels were associated with better survival and lower recurrence rates [140]. Another study involving 892 patients with stage II and III CRC indicated that low LMR (< 3.4) combined with high NLR (≥ 5.5) and high mean corpuscular volume (MCV) (≥ 80.5) before surgery was significantly associated with poor DFS after R0 resection [141]. However, data from early‐stage disease are mixed; in a study of 323 patients with stage I gastric cancer (GC) and 152 stage I CRC patients undergoing surgery, low LMR (< 4.2) showed a poorer OS in GC but not significantly in CRC [142]. Interestingly, low LMR also predicted a higher risk of mortality from infectious and vascular causes in early‐stage patients.

These findings are further supported by a meta‐analysis including data from 15 retrospective observational studies involving 11 783 patients, which confirmed high LMR as a consistent predictor of better survival across studies [130].

##### 3.3.3.3 Diagnostic Value

Despite the development of treatment strategies, a large number of patients at advanced stages still lack effective treatment options. Therefore, early diagnosis of CRC is crucial for improving patient prognosis [107]. In a study comparing two groups of patients, those with CC and those with benign colon masses (the control group), it was found that levels of CA‐19‐9, NLR, D‐dimer, and T‐lymphocytes in CC patients were significantly higher than in the control group. Moreover, higher levels of these markers were observed in patients with more advanced tumor stages [143]. Chen et al. found that NLR (> 2.4), RDW (> 12.58 fL), and PLR (> 103.7) were significantly elevated in CRC patients compared to controls, and their combined use improved diagnostic accuracy [144]. Similarly, Stojkovic Lalosevic et al. demonstrated that CRC patients across all TNM stages (I, II, III, and IV) had significantly higher values of NLR (≥ 2.15) and PLR (≥ 123) compared to the control group, with enhanced diagnostic efficacy when used together with mean platelet volume (MPV) [110]. Liu et al. also highlighted the additive diagnostic value of CEA (> 2.63) along with NLR (> 2.10), d‐NLR (> 1.73), and PLR (> 146.71) in differentiating CRC patients from controls [145]. A study conducted by Li et al. revealed that combining inflammatory markers with CEA improves diagnostic performance in both early‐ and late‐stage CRC. For instance, NLR (≥ 1.81), PLR (≥ 128.03), or LMR (≥ 4.61) assessed alongside CEA (≥ 3.30 ng/mL) significantly distinguished early from advanced CRC [107]. Yu et al. further indicated that LMR (cut‐off = 3.28), hemoglobin‐to‐platelet ratio (HPR) (cut‐off = 0.62), and CEA (ng/mL cut‐off = 3.08) could be useful for the differential diagnosis of CC versus benign colon tumors, providing the most reliable results when used in combination rather than alone [146]. Finally, Chen et al.'s evaluation of 1163 CRC patients undergoing surgical treatment revealed that NLR, PLR, and tumor markers like CEA, CA‐19‐9, and CA‐50 (cancer‐antigen 50) were significantly higher in the metastasis group. Combining these inflammatory indices with tumor markers improved the predictive accuracy for metastasis [147].

Although individual markers provide moderate diagnostic value, their combination, especially with tumor‐specific antigens, enhances sensitivity and specificity. These findings support the integration of inflammatory indices into early diagnostic strategies for CRC, potentially reducing reliance on invasive procedures.

### 3.4 Prostate Cancer

#### 3.4.1 Introduction

PCa is the fourth most common cancer and the eighth leading cause of cancer‐related death worldwide [148]. Risk factors for this disease include family history, race, age, obesity, and environmental factors such as tobacco smoke. Common prognostic factors for this disease include prostate‐specific antigen (PSA) levels, TNM stage, and Gleason score, along with other factors such as urinary function, comorbidities, and patient age [149]. Treatment options for localized prostate cancer include radical prostatectomy (RP), external beam radiation therapy (XRT), brachytherapy, and cryotherapy, while treatment for advanced prostate cancer includes hormone therapy and chemotherapy [150]. PSA, although widely used, lacks specificity and leads to unnecessary biopsies due to its limited positive predictive value [151]. Apart from PSA, other factors such as digital rectal examination (DRE) and transrectal prostate ultrasound can also indicate the likelihood of cancer and the need for biopsy [152].

#### 3.4.2 Subtypes and Classification

Histologically, nearly all prostate cancers (95%) are adenocarcinomas. The remaining 5% of cases include squamous cell carcinoma, signet‐ring carcinoma, transitional carcinoma, neuroendocrine carcinoma, and sarcoma, each with distinct clinical features and often a poorer prognosis [153].

#### 3.4.3 Clinical Utility of Hematological Markers

Inflammatory ratios such as NLR, PLR, and LMR offer a practical approach to assessing systemic immune status. In PCa, these markers have been evaluated for prognostic, predictive, and diagnostic implications, as summarized below.

##### 3.4.3.1 Prognostic Value

Typically, the prognosis of patients with PCa is predicted by TNM staging, PSA levels, and Gleason score. However, patients with the same stage can exhibit different prognoses, and patients with varying PSA levels can show similar prognoses. Therefore, biomarkers with sufficient accuracy are needed to evaluate prognosis and make decisions regarding the necessary treatment strategies [154].

###### 3.4.3.1.1 NLR

Several studies have examined the relationship between NLR and PSA levels. Wang et al. reported that high NLR (> 3.3) was significantly and directly associated with higher PSA levels and advanced stages of PCa [155]. However, conflicting evidence from Salah et al. in patients with metastatic castration‐sensitive prostate cancer (mCSPC) revealed that although NLR ≥ 4 is an independent factor for lower OS, it doesn't correlate with PSA progression, suggesting that NLR may be more reflective of systemic inflammation than tumor burden [156].

Chemotherapy: Numerous studies have explored the prognostic role of NLR in patients with metastatic castration‐resistant prostate cancer (mCRPC), often reporting similar findings. For instance, in a 2022 study of patients treated with cabazitaxel after receiving docetaxel, it was demonstrated that high NLR (≥ 3) after treatment was directly associated with lower OS. Although it was not predictive of PFS or treatment response [157]. In a similar study, the correlation between NLR and both OS and PFS was evaluated in 41 mCRPC patients treated with docetaxel, revealing that high NLR (> 3) before treatment could independently predict lower PFS and OS [158].

Androgen replacement therapy: Androgen replacement therapy (ART), which includes treatment with enzalutamide (ENZ) or abiraterone acetate (ABI), is another treatment option for mCRPC patients. In a retrospective study of 449 patients who received first‐line ART, an NLR ≥ 3.02 was associated with inferior survival outcomes [159]. Moreover, while a high NLR (≥ 3) again correlated with shorter PFS, a study involving 225 mCRPC patients treated with ENZ or ABI found no significant association between baseline PLR and prognosis, further highlighting the stronger prognostic reliability of NLR in this setting [160].

Surgery: As mentioned, RP is considered the gold standard for the treatment of localized PCa. Some studies have associated biochemical recurrence (BCR) after RP with histopathological factors [161, 162]. In a study involving 668 patients with localized PCa who underwent RP, no significant association was observed between pre‐treatment NLR and key histopathological parameters or long‐term oncologic outcomes. This lack of prognostic value may be partially explained by the biology of early‐stage tumors, where lower systemic inflammation and higher intratumoral lymphocyte activity (reflected by a lower NLR) are common. In early disease, TILs play a critical role in cancer cell eradication, theoretically leading to a lower NLR that is less variable and less predictive of outcome compared to advanced cases [163].

###### 3.4.3.1.2 PLR

Hormone therapy: An analysis of 290 PCa patients undergoing first‐line androgen deprivation therapy (ADT) was conducted; high PLR (≥ 117.58) could predict worse PFS, CSS, and OS [164]. Notably, the inclusion of PLR in a conventional prognostic model (based on Gleason score and metastatic status) improved the predictive accuracy for survival outcomes. Similarly, Lozano Martínez et al. studied 101 patients with mCRPC treated with abiraterone; low PLR (< 150) and NLR (< 5) were significantly associated with better OS and PFS, while no correlation was observed between these markers and PSA response [165]. However, the lack of association with biochemical response suggests these markers should complement, rather than replace, traditional indicators in clinical decision‐making.

Radiotherapy: In a cohort of 374 PCa patients undergoing radiotherapy, PLR ≥ 190 was a prognostic factor for reduced MFS, CSS, and OS, and could be helpful in oncological treatment decisions [166].

Surgery: In a study involving 440 high‐risk localized PCa patients who underwent RP, high d‐NLR (cut‐off = 1.3), high PLR (cut‐off = 100.7), and low PNI (cut‐off = 47.4) were associated with poor biochemical disease‐free survival [167], suggesting the potential benefit of combining inflammatory and nutritional markers for individualized risk stratification.

###### 3.4.3.1.3 LMR

Radiotherapy: In a large cross‐sectional analysis of 7706 men from the NHANES database, a significant inverse association was found between LMR and PCa risk, with a threshold effect at LMR = 4.86. While this suggests that higher LMR may be protective, the study's observational design limits causal inference [168]. In a multicenter study of 519 mCRPC treated with radium‐233 dichloride, the prognostic value of the three indices, including NLR, PLR, and LMR, was incorporated into a composite BIO‐Ra score. Although LMR alone was not predictive of treatment response (e.g., ALP decline), high LMR (cut‐off = 2.8) predicted higher OS [169]. These results suggest that combining LMR with other markers may enhance prognostic accuracy in radium therapy. In contrast, a smaller study of 152 PCa patients treated with radiotherapy revealed that higher LMR (> 3.26) values before treatment were directly associated with worse OS. It was also noted that an increase in PLR (> 89.6) before treatment was statistically close to worse OS [170]. This result may reflect disease heterogeneity or altered inflammation during radiotherapy. As only the lymphocyte count correlated with PSA, LMR's prognostic value appears limited, supporting the need for multimodal biomarker approaches.

##### 3.4.3.2 Predictive Value

Several studies have examined the predictive value of circulating immune cell ratios in PCa. LMR, PLR, and NLR have all been associated with clinical outcomes in most of these studies.

###### 3.4.3.2.1 NLR

Brachytherapy: Managing low‐risk and intermediate‐risk PCa can be challenging due to the heterogeneous nature of these cancers. In one study, patients with low‐risk and intermediate‐risk PCa were all treated with brachytherapy. It was observed that patients with NLR > 3 had lower rates of BCR, suggesting that NLR could serve as a useful marker for predicting disease progression and treatment response [171].

Surgery: Approximately 30%–50% of localized PCa patients who undergo RP may develop BCR, which could be closely related to tumor recurrence and metastasis. Wang et al. demonstrated that a high NLR (≥ 2.62) measured prior to surgery had a direct association with higher BCR rates and lower BCR‐free survival following RP in 114 localized PCa patients [172].

Conversely, Bravi et al. reached a different conclusion in a retrospective study involving a larger group of localized PCa patients (1258 patients) in 2022, finding that although NLR was associated with BCR at the univariate level, this was not sustained in multivariable analysis [173]. The authors highlighted several factors (short follow‐up and lack of standard NLR cut‐off) that may explain these discrepancies and limit NLR's clinical reliability.

###### 3.4.3.2.2 PLR

Surgery: Ferro et al. evaluated 260 PCa patients with clinically low‐risk PCa undergoing RP. They found that NLR, PLR, and eosinophil‐to‐lymphocyte ratio (ELR) are predictors of Gleason upgrading. Thus, these markers could be valuable in assessing low‐risk PCa [174]. However, Lee et al. analyzed PLR before prostate biopsy in a large cohort and found no independent association with clinically significant cancer, indicating limited predictive use of PLR for biopsy decision‐making [175].

###### 3.4.3.2.3 LMR

Hormone therapy: In a secondary analysis of the LATITUDE trial, the prognostic value of dynamic changes in PSA, NLR, PLR, LMR, and hemoglobin over 24 months was assessed in high‐risk, de novo metastatic, hormone‐sensitive prostate cancer patients treated with ADT plus abiraterone or placebo. A multivariate joint model showed that rising PSA and changes in these inflammatory markers were associated with inferior OS and had clinical benefit over baseline‐only models. These findings suggest that longitudinal monitoring of serologic markers may help identify individuals who are likely to have an unfavorable outcome early in the disease course [176].

##### 3.4.3.3 Diagnostic Value

While PSA remains the most commonly used tool for diagnosing and predicting PCa, its limited specificity and sensitivity have prompted research into additional diagnostic markers. In a study conducted in 2022 to evaluate the value of NLR in diagnosing and differentiating localized PCa from other conditions such as benign prostatic hyperplasia (BPH), NLR showed significant correlations with tumor aggressiveness markers: it was higher in patients with PSA > 10 ng/mL, in those with Gleason Score ≥ 7, and in patients with extracapsular extension (pT3) and lymph node involvement. These findings suggest that although NLR has limited diagnostic value for early detection, it may still serve as a supportive indicator for tumor progression and nodal metastasis in localized PCa [177]. In contrast, Wang et al. conducted a study with a smaller patient population (117 patients) and found that, compared to patients with BPH, PCa patients exhibited higher levels of SII (≥ 471.86), neutrophil percentage (≥ 65.15%), and NLR (≥ 1.6), suggesting their potential diagnostic utility. However, a combination of these markers with total PSA did not substantially improve diagnostic accuracy [152]. The advantage of identifying an efficient marker for diagnosing PCa is that it allows patients to recognize their risk of cancer more quickly and receive timely medical intervention. To this end, a study in China evaluated 378 men with PSA levels below 10 ng/mL during their first biopsy and concluded that a high NLR enhances the diagnosis of PCa, suggesting its possible use as a complementary diagnostic tool in patients with lower PSA levels [178].

## 4 Summary of Evidence and Critical Appraisal

Elevated levels of NLR and PLR, along with decreased LMR, are frequently associated with poorer prognosis, shorter survival, and higher recurrence rates across various cancers [93, 94, 127]. These markers reflect a systemic inflammatory response that may contribute to tumor growth and metastasis. Moreover, they have potential utility in predicting patient response to different treatment modalities such as chemotherapy, radiotherapy, or immunotherapy [64, 95]. Although all four cancers have a substantial body of evidence and meta‐analyses, the focus of studies varies: lung cancer research emphasizes predicting response to immunotherapy, BC studies focus on triple‐negative and HER2‐positive subtypes, CRC research primarily addresses recurrence prediction after surgery, and PCa studies mainly concentrate on its role in CRPC. In some studies, combining NLR, PLR, and LMR with other biomarkers such as CA‐50, CA‐19‐9, CEA, HPR, RDW, or MPV [67, 110, 146, 147], or with each other [14], has enhanced their diagnostic and prognostic performance. Some findings also suggest that dynamic changes in these biomarkers, rather than baseline values alone, may provide stronger prognostic value for survival [49]. Some studies have shown that NLR may have a stronger prognostic value compared to PLR or LMR [50, 52, 160], whereas others have reported the opposite [55, 93, 94]. However, several studies have reported no prognostic significance for these ratios [83, 89, 97, 142]. Currently, classical inflammatory markers such as CRP and ESR are widely used in clinical practice for monitoring the severity of inflammation in conditions like infections and postoperative care, and they hold an established role in patient management [179, 180]. In contrast, indices such as NLR, PLR, and LMR are largely confined to research and specific prognostic or adjunctive applications, with their position in routine clinical practice still evolving. In oncology, NLR, PLR, and LMR have not yet been incorporated into formal guidelines [181, 182, 183, 184, 185] and are only applied to a limited extent alongside treatment to estimate therapeutic response. Classical parameters such as TNM staging, tumor markers, and PD‐L1 expression remain the primary clinical standards [186, 187, 188]. Although NLR, PLR, and LMR have demonstrated potential as cost‐effective and readily accessible biomarkers, their adoption in the routine management of common solid tumors remains limited. The primary reason for this limited acceptance is the lack of strong, comprehensive, and consistent evidence. Most existing studies are retrospective, single‐center, and limited by methodological issues such as insufficient clinical and molecular data, reliance on cross‐sectional designs, and unmeasured confounding factors [89, 173]. In addition, there is no universally established standard for cut‐off values [88, 89]. Furthermore, no prospective multi‐center trials have established validated thresholds or demonstrated superiority over standard biomarkers.

Future research should be prospective, incorporate precise molecular subtyping and clinical staging, and control for key confounders such as CRP, hormone levels, and long‐term medication use. They should include longitudinal measurements to capture the dynamic nature of inflammation and determine validated, optimal cut‐off values. Research must also address subgroup‐specific differences and enroll large, balanced cohorts to improve statistical power and generalizability. Finally, external validation across diverse populations and efforts to minimize heterogeneity are essential to support clinical implementation.

## 5 Conclusion

Inflammatory indices derived from complete blood counts, including NLR, PLR, and LMR, which can be easily obtained from a blood test as part of routine clinical care, have demonstrated potential value in predicting prognosis, treatment responses, and diagnosis for patients with breast, lung, colorectal, and prostate cancers. Their ease of measurement, low cost, and reproducibility make them attractive candidates for clinical application. However, while many studies support their prognostic significance, evidence for their diagnostic and predictive utility remains inconsistent, underscoring the need for standardized thresholds and further validation through well‐designed prospective trials.

## Author Contributions

**Fatemeh Arianmanesh:** writing – original draft. **Saeede Bagheri:** writing – review and editing. **Mohammad Ali Karimi:** writing – original draft. **Saba Izadi:** writing – original draft. **Mohammad Hossein Ahmadi:** writing – review and editing, supervision.

## Funding

The authors have nothing to report.

## Ethics Statement

The authors have nothing to report.

## Conflicts of Interest

The authors declare no conflicts of interest.

## Data Availability

Data sharing is not applicable to this article as no new data were created or analyzed in this study.
